# Association between abnormal default mode network activity and suicidality in depressed adolescents

**DOI:** 10.1186/s12888-016-1047-7

**Published:** 2016-09-29

**Authors:** Shuang Zhang, Jian-mei Chen, Li Kuang, Jun Cao, Han Zhang, Ming Ai, Wo Wang, Shu-dong Zhang, Su-ya Wang, Shi-jing Liu, Wei-dong Fang

**Affiliations:** 1 Mental Health Center, University-Town Hospital of Chongqing Medical University, Chongqing, 401331 China; 2 Department of Psychiatry, First Affiliated Hospital of Chongqing Medical University, Chongqing, 400016 China; 3 Center for Cognition and Brain Disorders and the Affiliated Hospital, Hangzhou Normal University, Hangzhou, 310015 China; 4 Department of Radiology, First Affiliated Hospital of Chongqing Medical University, Chongqing, 400016 China

**Keywords:** Adolescents, Attempted suicide, Default mode network, Depression, rs-fMRI

## Abstract

**Background:**

Suicide is the second leading cause of death among 15- to 29-year-olds in China, and 60 % of suicidal patients have a history of depression. Previous brain imaging studies have shown that depression and suicide may be associated with abnormal activity in default mode network (DMN) regions. However, no study has specifically investigated the relationship between DMN functional activity and suicidal behavior in depressed individuals. Therefore, in the present study, we directly investigated features of DMN brain activity in adolescent patients with histories of depression and attempted suicide.

**Methods:**

A total of 35 sex- and age-matched suicidal depressed patients were compared with 18 non-suicidal depressed patients and 47 healthy controls. We explored functional activity changes in DMN regions that could be associated with suicidal behavior by comparing resting-state functional magnetic resonance imaging (rs-fMRI) signals using independent component analysis (ICA). Scores on six clinical scales that measure depression severity (Hamilton Depression Scale (HDRS) and Beck Depression Inventory (BDI)) and suicidal traits (Barratt Impulsiveness Scale (BIS-11), Suicide Attitude Questionnaire (SAQ), Beck Hopelessness Scale (BHS), and Scale for Suicide Ideation (SSI)) were compared in the three groups.

**Results:**

Compared with the healthy controls, all of the evaluated depressed patients showed increased functional connectivity in select DMN regions. The suicidal patients showed increased connectivity in the left cerebellum and decreased connectivity in the right posterior cingulate cortex (PCC), whereas the non-suicidal depressed patients showed increased connectivity in the left superior frontal gyrus, left lingual gyrus and right precuneus and decreased connectivity in the left cerebellum. Compared to the non-suicidal patients, the suicidal patients showed increased connectivity in the left cerebellum and the left lingual gyrus and decreased connectivity in the right precuneus. No differences in the scores of any clinical scales were found between the suicidal and non-suicidal depressed patients.

**Conclusions:**

Collectively, our results highlight the importance of the DMN in the pathophysiology of depression and suggest that suicidal behavior in depressed adolescents may be related to abnormal functional connectivity in the DMN. In particular, abnormal connectivity in the PCC/precuneus and left cerebellum might be a predictor of suicidal behavior in depressed adolescent patients.

## Background

Suicide is a serious and complex public health problem worldwide [1]. Adolescence is a time of high risk for suicidal behavior and is the best time for intervention and treatment [2]. Published studies indicate that up to 6 % of adolescents report attempting suicide in the previous year [3]. Suicidal behavior is closely related to depression, and depressed patients have a high lifetime risk of death by suicide [4]. Although suicidal behavior does not need to be present for a patient to meet the criteria for major depressive disorder (MDD), a history of suicide attempts may indicate a more severe course of the disorder [5]. Previous research has found a 6-fold higher suicide risk in the offspring of patients with mood disorders who have attempted suicide relative to the offspring of patients with mood disorders who have not attempted suicide [6], suggesting that a close relationship exists between suicidal behavior and depression. However, it is notable that accumulating evidence has also shown that suicidal behavior may exist independently of mood disorders [4, 6–9]. Therefore, the mechanism underlying the progression to suicide in patients with depression remains unclear. In magnetic resonance imaging (MRI) studies, depression has been associated with structural and functional abnormalities within select brain regions, such as the prefrontal cortical region and limbic region, both of which have been implicated in the regulation and processing of emotions [10, 11]. The default mode network (DMN) has been suggested to participate in the processes of introspection and emotion [12], and depression is known to involve the pathological inability of the DMN to regulate self-referential activity in a situationally appropriate manner [12–15]. Furthermore, connectivity changes in the DMN have been correlated with pessimism during depressive episodes, but not with treatment response [16].

Riachle first proposed the concept of the DMN, which is primarily composed of the dorsal and ventral medial prefrontal cortices, the medial and lateral temporal cortices, the medial and lateral parietal cortex and the precuneus/posterior cingulate cortex (Pc/PCC) [12, 17–19]. DMN activity decreases when an individual expends attention on behavioral assignments and increases when an individual is not working on specific cognitive tasks [20–22]. High functional connectivity within the DMN and failure to down-regulate DMN activity during participation in goal-directed tasks can distinguish depressed patients from normal subjects [12, 13]. Previous studies have suggested that alterations in DMN activity in depressed patients primarily occur in the medial prefrontal cortex (MPFC), anterior cingulate cortex (ACC), PCC, precuneus, orbito-frontal cortex and hippocampus [23–25]. Using voxel-based morphometry (VBM) and functional connectivity analysis, significant gray matter abnormalities in the frontal gyrus, hippocampus/amygdala, right middle temporal gyrus (MTG) and bilateral caudate have been previously found in depressed patients, as have DMN connectivity alterations [26, 27]. Although numerous MRI studies have been conducted to assess the relationships that exist between different brain networks in patients with depression [28], there is a paucity of studies regarding adolescent depression and its association with attempted suicide. Structural MRI studies of the brain have revealed gray matter and white matter abnormalities in suicidal depressed patients [29], and reductions in gray matter volume in the ACC, prefrontal cortex, parietal-occipital cortex, orbitofrontal cortex, caudate, insula and cerebellum as well as increases in gray matter volume in the right amygdala have been identified in depressed patients with suicidal behavior relative to non-suicidal depressed patients [26, 30, 31]. White matter abnormalities are associated with cognitive impairment, and young patients with depression who have attempted suicide have more prevalent white matter hyperintensities (WMHs) and white matter lesions (WHLs) at baseline [5, 30, 32]. Although these studies suggest a probable relationship between suicidal behavior in depression and some regions of the DMN, no study has examined the DMN in adolescents with depression who have attempted suicide. Based on previous findings, we hypothesized that functional activity alterations in DMN regions such as the frontal gyrus, temporal gyrus, PCC and precuneus are present in suicidal depressed adolescents.

Blood oxygen level-dependent (BOLD) functional MRI (fMRI) is a promising neuroimaging technique that can noninvasively measure intrinsic and spontaneous neural activity fluctuations that reflect the oscillations of disparate neural networks at synchronized frequencies [33]. Compared with the conventional region-of-interest (ROI) seed-based correlation method, independent components analysis (ICA) is a data-driven blind source separation approach that aims to separate spatially or temporally independent patterns. This type of analysis thus allows model-free analysis of whole-brain fMRI data without the seed region selection and placement that are inherent to analyses of functional connectivity [33, 34]. ICA is also able to extract noise from a desired dataset and is thus a powerful method for detecting interactions within network regions [34]. Therefore, the current study was designed to test our hypothesis that functional activity alterations in DMN regions are present in suicidal depressed adolescents by using ICA to compare DMN activity changes in resting-state fMRI (rs-fMRI) signals between adolescents with depression who have attempted suicide, non-suicidal adolescents with depression and healthy controls (HCs). To the best of our knowledge, this is the first study to investigate what correlations exist between DMN functional connectivity alterations and suicide attempts in depressed adolescents using ICA.

## Methods

### Subjects

All study participants were Chinese Han people. Fifty-three patients with depression who were between 15 and 25 years old were recruited from the inpatient and outpatient units of the First Affiliated Hospital of Chongqing Medical University and University-Town Hospital of Chongqing Medical University from April 2012 to December 2013. The patient cohort was divided into two groups: a suicidal depressed group (SD group) composed of 35 patients with depression and a history of at least one suicide attempt and a non-suicidal depressed group (NSD group) composed of 18 patients with depression and no history of attempted suicide. A suicide attempt was defined as a self-injurious act associated with at least some intent to die as a result of the act, regardless of whether the attempt resulted in actual injury. Evidence that an individual intended to kill him/herself could be explicit or inferred from the individual’s behavior or circumstances [35]. The Structured Clinical Interview for DSM-IV-TR Axis I disorders (SCID-I/P) was used to identify adolescents with depressive symptoms and to develop a healthy control (HC) group [36]. The enrolled suicidal and non-suicidal depressed patients refrained from the use of antidepressants for at least 2 weeks prior to the start of the study and from the use of electroconvulsive therapy for at least 4 weeks prior to the start of the study. A total of 47 sex- and age-matched individuals were recruited from the local community to serve as the HC group. The following exclusion criteria were employed: history of neurological illness; severe traumatic brain injury; current or previous psychiatric disorder other than depression; heart, liver, or kidney disease or other serious physical illness; any type of contraindication for MRI; and substance dependence or abuse. The Intelligence Quotient (IQ) of each subject was measured using the Chinese Combined Raven’s Test, Copyright 2 (CTR-C2) [37]. The Hamilton Depression Rating Scale (HDRS, [38]) and Beck Depression Inventory (BDI, [39]) were used to evaluate depression severity. The Suicide Attitude Questionnaire (SAQ, [40]), a 29-item-long self-reported checklist, was used to describe attitudes related to suicide along 4 dimensions: (1) view of suicidal behavior, (2) attitude towards suicide survivors, (3) family history of suicide attempts, and (4) perspective on euthanasia. The Scale for Suicide Ideation (SSI, [41]) was also employed; higher scores on this scale indicate a stronger intention to commit suicide. The Barratt Impulsiveness Scale-11 (BIS-11, [42]) was used to measure impulsive personality traits, and the Beck Hopelessness Scale (BHS, [43]) was used to measure negative attitudes about the future. All subjects personally agreed to participate in this study. Subjects who were older than 18 years signed written informed consent before the experiment. For subjects younger than 18 years, our group contacted family members to obtain written informed consent from the subjects’ legal guardians. The study was approved by the Ethics Committee of the First Affiliated Hospital of Chongqing Medical University in China.

### Image acquisition

Images were acquired using a GE Signa Hdxt 3.0-Tesla scanner (General Electric Medical Systems, Milwaukee, WI, USA) with a standard eight-channel head coil; the scanner was located at the First Affiliated Hospital of Chongqing Medical University. Foam padding was used to minimize head motion and machine noise. Each participant was instructed to lie down and relax, to keep their eyes closed, to stay awake and to avoid performing specific cognitive tasks. Conventional T1-weighted images (using fast spin echo (FSE), repetition time/echo time (TR/TE) = 500 ms/14 ms, thickness/gap = 5.0/0 mm, flip angle = 45°, NEX = 1, field of view (FOV) = 24×24 cm, and matrix = 256×126) and high-resolution 3D T1 images (using fast gradient echo (FGRE), TR/TE = 24 ms/9 ms, flip angle = 90°, thickness/gap = 1.0/0 mm, FOV = 24×24 cm, and matrix = 256×256) were acquired. rs-fMRI images were obtained using an echo-planar image (EPI) pulse sequence with the following parameters: 33 axial slices, TR/TE = 2000/40 ms, matrix = 64×64, flip angle = 90°, FOV = 240×240 mm^2^, thickness/gap = 4.0/0 mm. A total of 240 time points were axially recorded over 8 min. No obvious structural damage was found, and none of the subjects felt discomfort during or after the procedure.

### Image preprocessing and DMN identification

All resting-state functional images were analyzed using Data Processing Assistant for Resting-State fMRI Advanced Edition (DPARSF; http://www.restfmri.net) [44] and the REST 1.9 (http://www.restfmri.net) [45] toolbox in Matlab version 7.8.0 (MathWorks, Inc., CA). The first 10 time points were discarded to allow for scanner calibration and participant adaptation to the scanning environment. The preprocessing steps included slice timing, head motion correction, spatial normalization in Montreal Neurological Institute (MNI) space (resampling with 3 × 3 mm3 resolution), smoothing (a smoothing kernel with FWHM of 4x4x4 mm^3^) [46], linear trend removal and filtering (0.01–0.08 Hz). Six parameters associated with head motion signals were regressed out (3 for shift and 3 for rotation), and the overall head motion was calculated as the average magnitude of the head motion. No significant differences were found in the root mean square (RMS) head movement among the three groups (*p* = 0.35). There was less than 1 mm maximum displacement in subject head movement in any direction (x, y, and z) and less than 1° maximum displacement in any angular dimension. The preprocessed images of each subject were subjected to group ICA to identify functional networks and their temporal activities using a MICA toolbox (http://www.nitrc.Org/projects/cogicat) [47]. Three-stage principal component analysis (PCA) was used to reduce the dimensionality of the data to 40. The Infomax algorithm [48] was used for ICA decomposition; this analysis was repeated 100 times to achieve robust and accurate results. Then, spatially independent components (ICs) were back-reconstructed for each subject, and subject-specific spatial maps and time courses were acquired and z-score transformed. The ICs were grouped into different brain networks of interest, including the DMN, based on visual inspection, previous investigations and reported spatial patterns [49, 50].

### Statistical analysis

Statistical analyses of clinical and demographic subject characteristics were conducted using SPSS 17.0 software. One-way analysis of variance (one-way ANOVA) was used to evaluate group differences in age, years of education, IQ scores, and scores on the six included clinical scales. Post hoc pairwise comparisons were performed using least significant difference (LSD) t tests. Gender differences among the three groups were assessed using the chi-squared test. The brain rs-fMRI maps of the three groups were analyzed using REST 1.9 software. First, to characterize a selected DMN, individual spatial maps from the SD, NSD and HC groups were separately subjected to a group-specific one-sample *t*-test with AlphaSim multiple comparisons correction within a whole-brain mask using a voxel-wise threshold of *p* < 0.05 and a cluster threshold of ≥85 voxels (1000 Monte Carlo simulations) [51]. Then, the brain regions significantly connected within the DMN at the group level in the three groups were combined and investigated using one-way ANOVA for between-group comparisons, which revealed whether there were several regions in which there was a main effect or group difference. Clusters >324 mm^3^ (12 voxels) with *p* < 0.01 (as determined by multiple comparisons correction using the AlphaSim tool in the REST software package with rmm = 5) were deemed potential ROIs. The resultant ROIs were used to create a mask for further analysis. To further reveal the directions of the group differences, post hoc t-tests were applied to compare connectivity differences in the mask between any two groups. Post hoc two-sample t-tests were restricted to the voxels showing significant differences by one-way ANOVA. Subregions with corrected *p* < 0.01 and a cluster size >324 mm^3^ (12 voxels) were considered significantly different between groups. In the case of type I error, we extracted the average time course of each ROI in the mask for the three groups and compared them using one-way ANOVA and post hoc pairwise comparisons (LSD t-tests) (*p* < 0.01).

## Results

### Clinical and demographic subject characteristics

One-way ANOVA and chi-squared tests showed that the scores on all six clinical scales significantly differed among the three groups (all *p* < 0.001) (Table 1). There were no differences in gender, age, educational level or IQ score. Post hoc analysis showed that the scores on all six scales significantly differed between the HCs and the depressed patients with and without suicide history (*p* < 0.001), whereas no differences were found between the depressed patients with and without suicide history (see Table 1).

**Table 1:** Clinical and demographic characteristics for the three groups

Variable			Depression with		Depression without		F/*P* value	Contrast
	HC (*n* = 47)		Sui (*n* = 35)		Sui (*n* = 18)			
	Mean	SD	Mean	SD	Mean	SD		
Age (years)	20.48	1.86	20.63	3.65	21.26	3.02	0.584	
Gender (male/female)	16/31		10/25		8/10		0.602	
Education (years)	13.54	1.5	12.97	1.9	13.21	1.4	0.289	
IQ	100.87	16.45	100.02	18.23	102.42	8.77	0.872	
BIS-11	61.69	8.13	73.17	10.05	68.37	8.56	<0.001	SD,NSD > HC
SAQ	70.67	9.83	80.86	9.21	77.42	10.48	<0.001	SD,NSD > HC
HDRS	3.83	2.35	20.03	5.61	23.47	8.67	<0.001	SD,NSD > HC
BDI	3.96	3.32	23.91	7.94	26.21	5.24	<0.001	SD,NSD > HC
BHS	3.72	2.41	10.86	3.93	9.11	3.96	<0.001	SD,NSD > HC
SSI	2.94	2.27	11.06	4.71	11.21	4.25	<0.001	SD,NSD > HC

*Abbreviations*: *HC* healthy control, *Depression with Sui* depression with suicide attempt, *Depression without Sui* depression without suicide attempt, *SD* standard deviation, *IQ* intelligence quotient, *BIS-11* Barratt Impulsiveness Scale-11, *SAQ* suicide attitude questionnaire, *HDRS* Hamilton Depression Scale, *BDI* Beck Depression Inventory, *BHS* Beck Hopelessness Scale, *SSI* Scale of Suicide Ideation

### Brain connectivity differences between the HCs and the depressed patients with and without suicide history

**Fig. 1:**
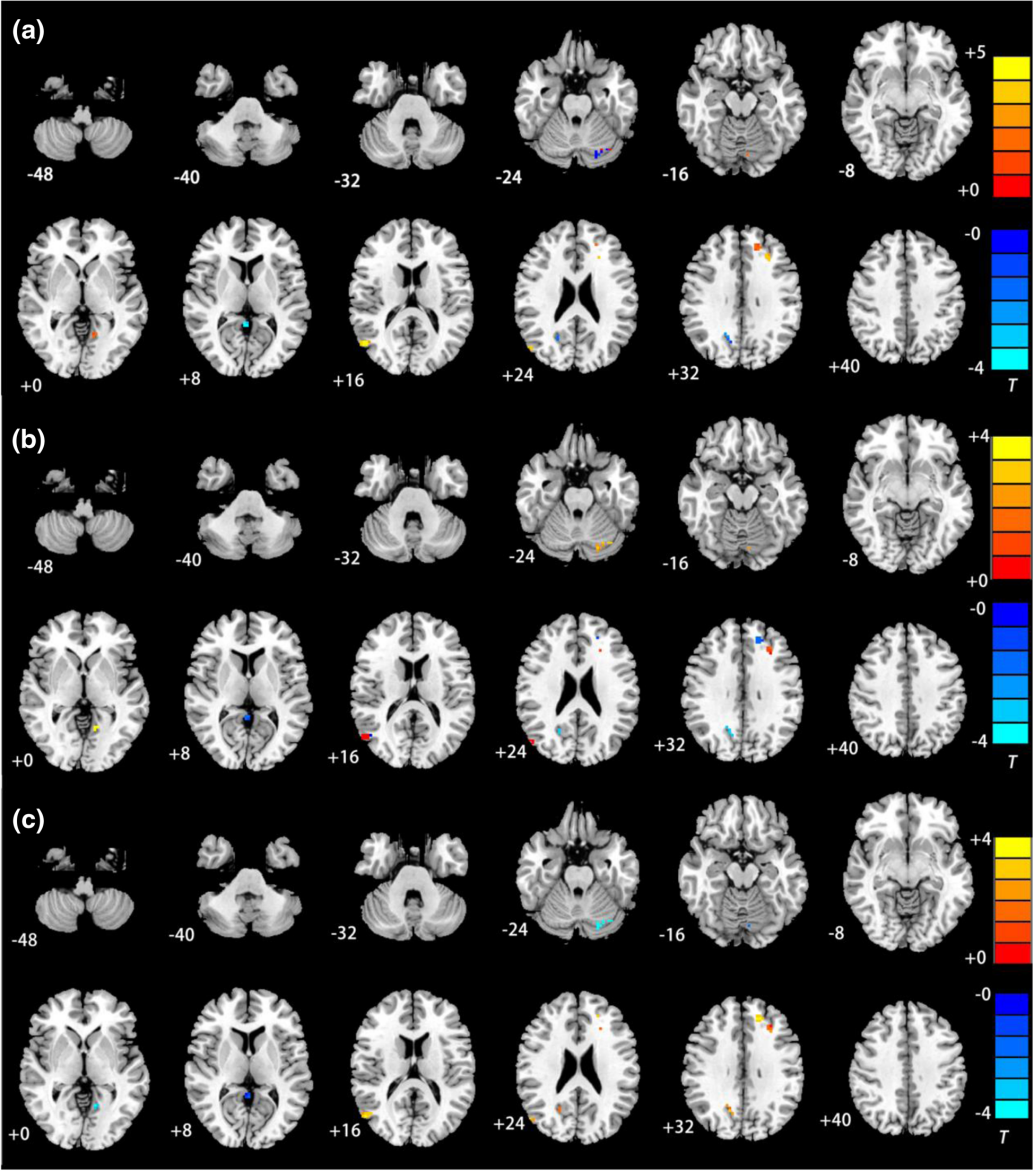
Between-group differences in DMN connectivity **a** Brain regions with aberrant connectivity in the SD group relative to the HC group. **b** Brain regions with aberrant connectivity in the SD group relative to the NSD group. **c** Brain regions with aberrant connectivity in the NSD group relative to the HC group. In all cases, the warm colors indicate increased connectivity and the cool colors indicate decreased connectivity. The numbers at the bottom left corner of each image refer to the z coordinates in Montreal Neurological Institute (MNI) space. The threshold was set at a corrected *p* < 0.01. T-score bars are shown at the right of each map. The left sides of the images correspond to the right side of the brain and vice versa

Compared with the HCs, the depressed patients with and without suicide history showed increased functional connectivity in the right MTG, right middle occipital gyrus and left middle frontal gyrus. Moreover, the suicidal patients showed increased connectivity in the left cerebellum [−6 −75 −18] and decreased connectivity in the right PCC [0 –48 3], whereas the non-suicidal depressed patients showed increased connectivity in the left superior frontal gyrus, left lingual gyrus, and right precuneus and decreased connectivity in the left cerebellum. Compared with the non-suicidal depressed patients, the suicidal group showed increased connectivity in the left cerebellum [−10 −75 −20] and left lingual gyrus [−13 −57 −1] and decreased connectivity in the right precuneus [18 −60 30] (see Fig. 1 and Table 2).

**Table 2:** Brain regions with ICA connectivity differences among the three groups

Brain region	Brodmann areas	MNI coordinates			*T* value	Cluster size (voxels)
	(BA)	X	Y	Z		
SD > HC						
R middle temporal gyrus	39	52	−69	17	4.7	30
R middle occipital gyrus	39	50	−72	25	3.31	30
L middle frontal gyrus	46	−30	27	33	3.56	15
L cerebellum	18	−6	−75	−18	2.42	15
SD < HC						
R posterior cingulate cortex	29	0	−48	3	−3.82	15
SD > NSD						
L cerebellum	18	−10	−75	−20	3.33	37
L lingual gyrus	18	−13	−57	−1	3.66	16
SD < NSD						
R precuneus	23	18	−60	30	−4.48	24
NSD > HC						
R middle temporal gyrus	39	51	−72	21	3.41	28
R middle occipital gyrus	39	53	−71	25	3.08	28
L superior frontal gyrus	32	−17	42	29	3.70	23
L middle frontal gyrus	32	−18	39	27	3.61	23
L lingual gyrus	18	−10	−56	−2	2.81	12
R precuneus	23	20	−59	26	2.35	22
NSD < HC						
L cerebellum	19	−26	−73	−23	−2.89	30

*Abbreviations*: *L* left, *R* right, *HC* healthy controls, *SD* suicidal depressed patients, *NSD* non-suicidal depressed patients, *ICA* independent component analysis, *MNI* Montreal Neurological Institute

## Discussion

In the current study, we found that suicidal and non-suicidal depressed patients showed increased functional connectivity in the right MTG, right occipital gyrus and left middle frontal gyrus compared with matched HCs. We also found increased connectivity in the left superior frontal gyrus, left lingual gyrus and right precuneus in the non-suicidal depressed patients. Collectively, these results suggest that functional connectivity in the DMN is broadly increased during depression. These results are partially consistent with the concept of “double dissociation”, in which depression is characterized by high resting functional connectivity within the DMN and low functional connectivity within the cognitive control network [16]. Furthermore, these findings support the hypothesis that the neuropathological mechanisms underlying depression primarily involve fronto-limbic circuitry [31, 52, 53]. The view that depression is strongly correlated with activity changes in the DMN at rest is widely accepted, although the use of functional neuroimaging to identify biomarkers of risk and the neural circuitry underlying the psychopathology of attempted suicide is a relatively young field.

As described above, compared with the HCs, the patients who had previously attempted suicide exhibited altered connectivity in the MTG, occipital gyrus and frontal gyrus. Suicidal behavior is associated with changes in the structural and functional characteristics of parietal-occipital-temporal areas [54]. Previous comparisons of structural and functional imaging studies of the suicidal brain have shown that the orbitofrontal and dorsolateral regions of the prefrontal cortex are the primary sites of alteration in suicidal patients [55]. Reduced activity in the ventromedial prefrontal cortex is associated with both increased suicidal intent and suicide attempts with high lethality [56]. High lethality suicide attempts require hospitalization to treat the resultant sequelae [57]. Suicide risk in individuals with major mental illnesses is directly associated with prefrontal cortex-based circuit dysfunction when engaging in cognitive tasks [58]. Additionally, hypo-connectivity from the left MPFC to the left PCC may be correlated with increased vulnerability to attempting suicide [59]. Vulnerability to suicidal behavior has also been associated with differences in responses to negative emotion. Marchand proposed that the striatum-anterior cortical midline structure (CMS) circuit, which is a component of the DMN, plays a significant role in the expression of depressive symptoms and suicidal ideation and that the striatum-motor/sensory cortex network may be a trait marker of suicide-related behavior [60, 61]. In short, we found that the depressed patients in this study who had attempted suicide exhibited functional connectivity changes in cortical regions. However, because the connectivity of the MTG, occipital gyrus and frontal gyrus in the non-suicidal and suicidal depressed patients was similar to that in the HCs and because no connectivity differences between the suicidal and non-suicidal depressed patients were found in these cortical structures, the importance of the connectivity of these structures in suicide is difficult to interpret.

In addition, our comparison of three groups showed that non-suicidal and suicidal depressed patients and HCs exhibited differences in the functional connectivity in the right PCC/precuneus. The PCC is a pivotal node in the DMN that mediates interactions between emotional and memory-related processes [62–64]. Prior imaging studies have indicated that the cingulate cortex may be a neuroanatomical correlate of suicidal behavior, as this region of the brain exhibits significant alterations in activation in depressed patients who have previously attempted suicide compared with psychiatric and healthy controls [65]. The precuneus plays a key role in the DMN through its engagement in a variety of processing states; connectivity between the precuneus and the DMN has been demonstrated to increase during rest [66]. The precuneus is also important for visuospatial imagery, episodic memory retrieval and self-processing operations [67]. Alexopoulos demonstrated that subjects suffering late-life depression had greater connectivity in the left precuneus [16], and precuneus hypoperfusion and white matter volume reduction were identified in suicidal patients [30, 68]. Our results for the PCC/precuneus were consistent with the above results, showing that depressed patients tend to have increased functional connectivity in the PCC/precuneus compared to HCs and that the functional connectivity in this region decreases as suicide ideation increases. Thus, the connectivity in this region might serve as a marker for the risk of suicide in depression. In the present study, we showed that functional connectivity in the left lingual gyrus differs between suicidal and non-suicidal depressed patients, although other imaging evidence that the left lingual gyrus may be involved in suicidal behavior in depression could not be found. More attention should be paid to the lingual gyrus during future studies of suicidal behavior.

As a special region of focus in the current study, we showed that functional connectivity in the left cerebellum particularly differed among the three groups: those who had previously attempted suicide exhibited higher connectivity than those who had not; however, the depressed patients showed decreased connectivity relative to the HCs. Previous studies have shown that the cerebellum not only affects balance and motor control but also cognition and emotional processing [69]. This region of the brain is involved in a variety of psychiatric disorders, including depression, bipolar disorder, and schizophrenia [70, 71]. In depressed patients, the cerebellum is typically smaller in size than in non-depressed patients and exhibits increased activity and disrupted cortical connections [69]. Decreased connectivity between the cerebellum and the DMN has also been found in patients with MDD [72, 73]. In a study reported by Phillips, different mental disorders were associated with abnormalities in different areas of the cerebellum. Reduced cerebellar volume should be further investigated in depressed patients. Compared to healthy subjects, suicidal patients have been shown to possess low regional cerebral blood flow in the cerebellum both at baseline and when concentrating [74]. In addition, suicidal depression has been associated with decreased gray matter and white matter volumes in the cerebellum compared to non-suicidal depression [30]. Individuals who have attempted suicide were also found to have increased impulsiveness and lower serotonin transporter (5-HTT) binding potential (BP) values, and 5-HTT BP was significantly correlated with integrity in the left cerebellar hemisphere [75]. An increasing number of studies about the suicidal brain have found the presence of biochemical abnormalities as well as structural and functional neuroimaging abnormalities in the cerebellum. The role of the cerebellum in mental illness and behavioral disorders involving cognition and emotional processing has been gradually recognized. Collectively, these results suggest that abnormal connectivity in the cerebellum may be a potential marker of suicidal behavior in depression.

### Limitations

This study requires replication and further verification in a larger patient population. We found no differences in total scores on the BIS, SSI, SAQ and BHS scales between the suicidal and non-suicidal depressed groups; this result may be due to the severity of the depression in the enrolled patients and the medical interventions the patients received before they were enrolled. Additional factors, including degree of depression, number of suicide attempts, family history of suicide, and medication load before enrollment, should be controlled in future studies because these factors may result in structural and functional DMN changes.

## Conclusions

In conclusion, the present study demonstrated that depressed patients exhibit a general increase in functional connectivity in the DMN. Distorted cognition often leads to negative beliefs and behaviors, and three cognitive characteristics (an attentional bias to particular life events reflecting signals of defeat, an insufficient capacity to solve problems and an absence of prospective anticipation of problems) can be differentiated between depressed suicidal individuals and depressed non-suicidal individuals. These characteristics are related to impairment in the DMN with regard to cognition and emotion. Suicide attempts in depressed adolescents may be related to abnormal functional connectivity in some DMN regions, and abnormal connectivity in the PCC/precuneus and left cerebellum may be predictors of suicide in depressed adolescents. The DMN represents a promising target for future neuroimaging studies to identify markers of risk for future suicide attempts in adolescents. The specific link between abnormal functional connectivity in the cerebellum and the PCC/precuneus, as measured by fMRI, and suicidal behavior in depressed patients should also be further investigated.

## Abbreviations

5-HTT: Serotonin transporter
ACC: Anterior cingulate cortex
BDI: Beck Depression Inventory
BHS: Beck Hopelessness Scale
BIS-11: Barratt Impulsiveness Scale
BOLD: Blood oxygenation level-dependent
BP: Binding potency
CMS: Cortical midline structure
DMN: Default mode network
HDRS: Hamilton Depression Scale
ICA: Independent component analysis
MPFC: Medial prefrontal cortex
MTG: Middle temporal gyrus
PCA: Principal component analysis
PCC: Posterior cingulate cortex
rs-fMRI: Resting-state functional magnetic resonance imaging
SAQ: Suicide Attitude Questionnaire
SCID: Structured Clinical Interview for DSM-IV Axis I disorders
SSI: Scale for Suicide Ideation
WHLs: White matter lesions
WMHs: White matter hyperintensities
